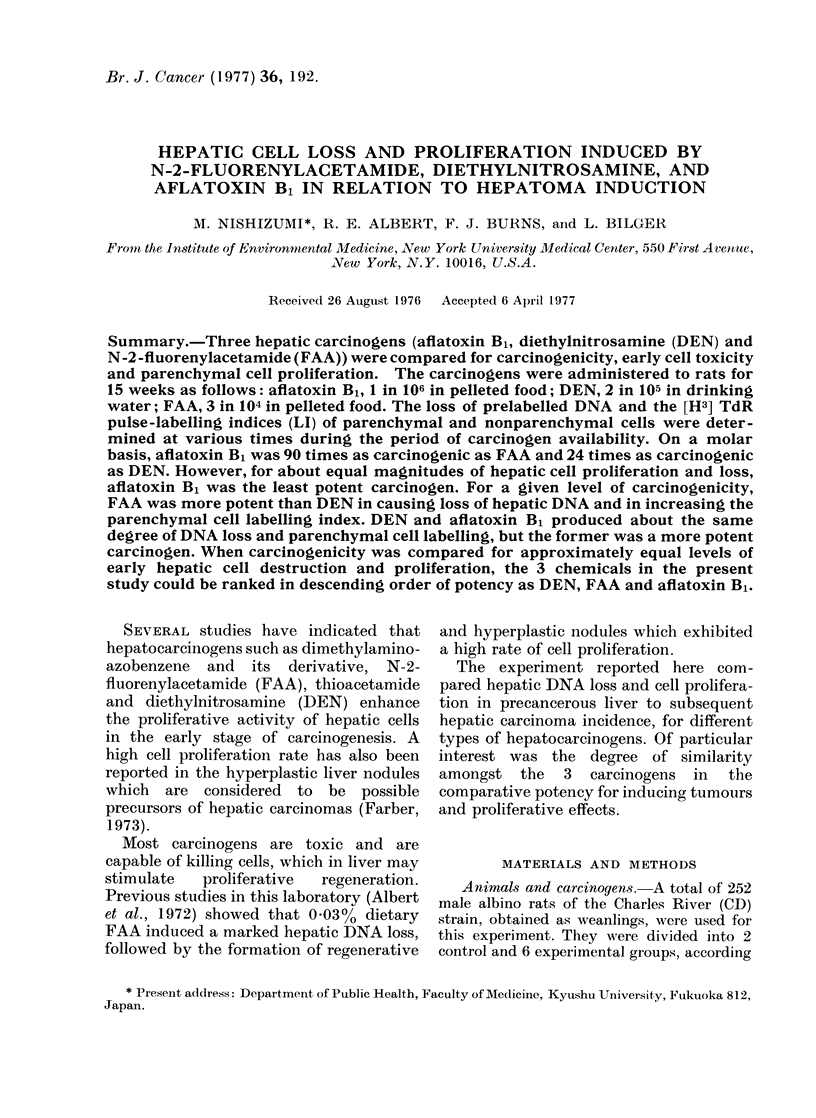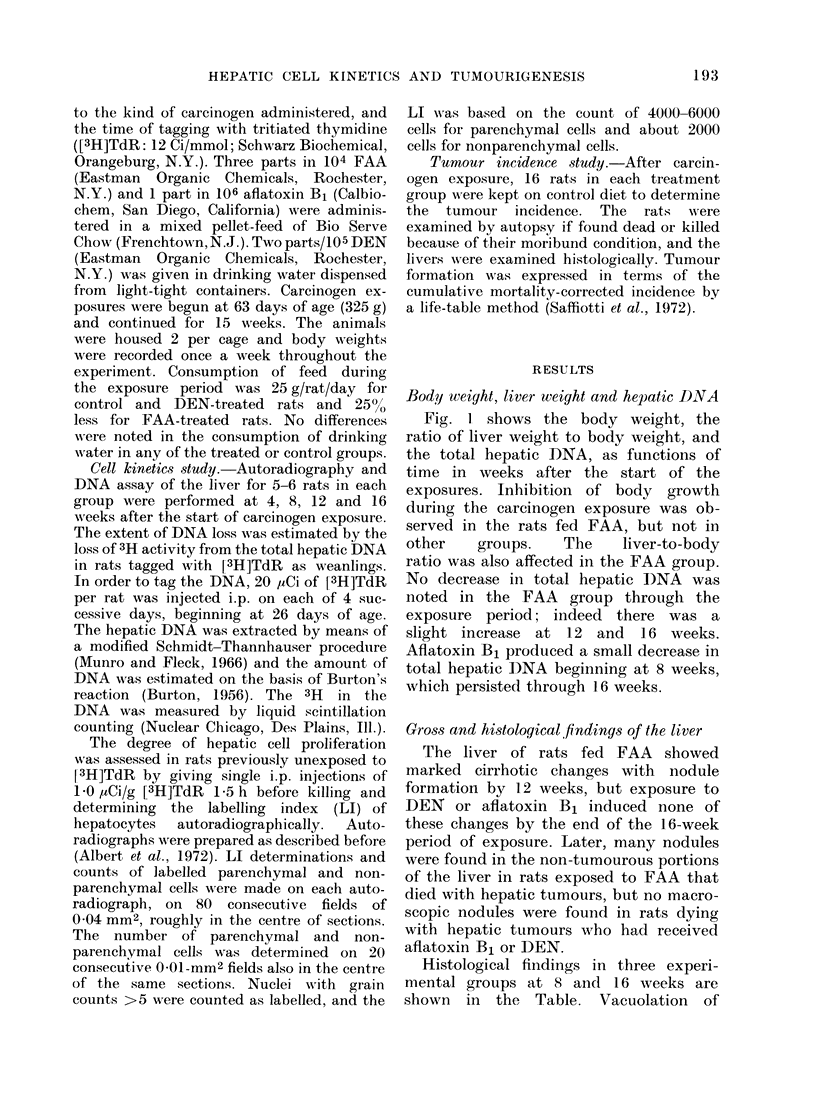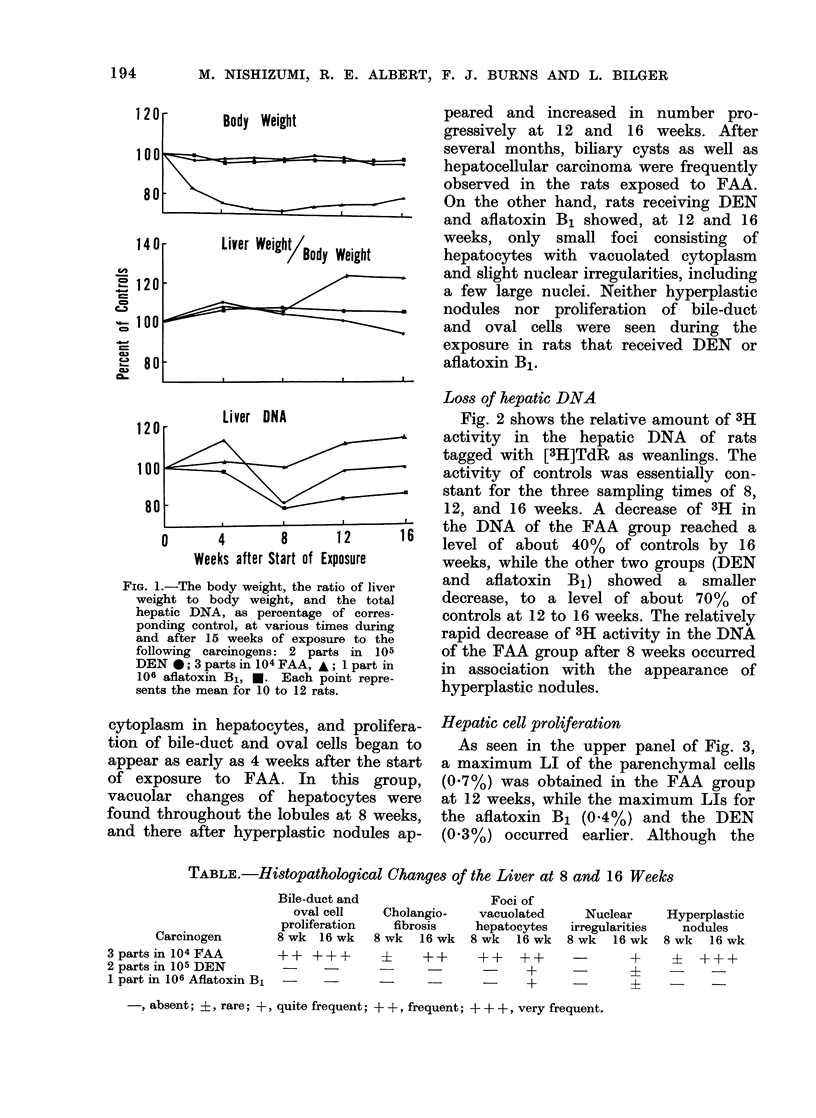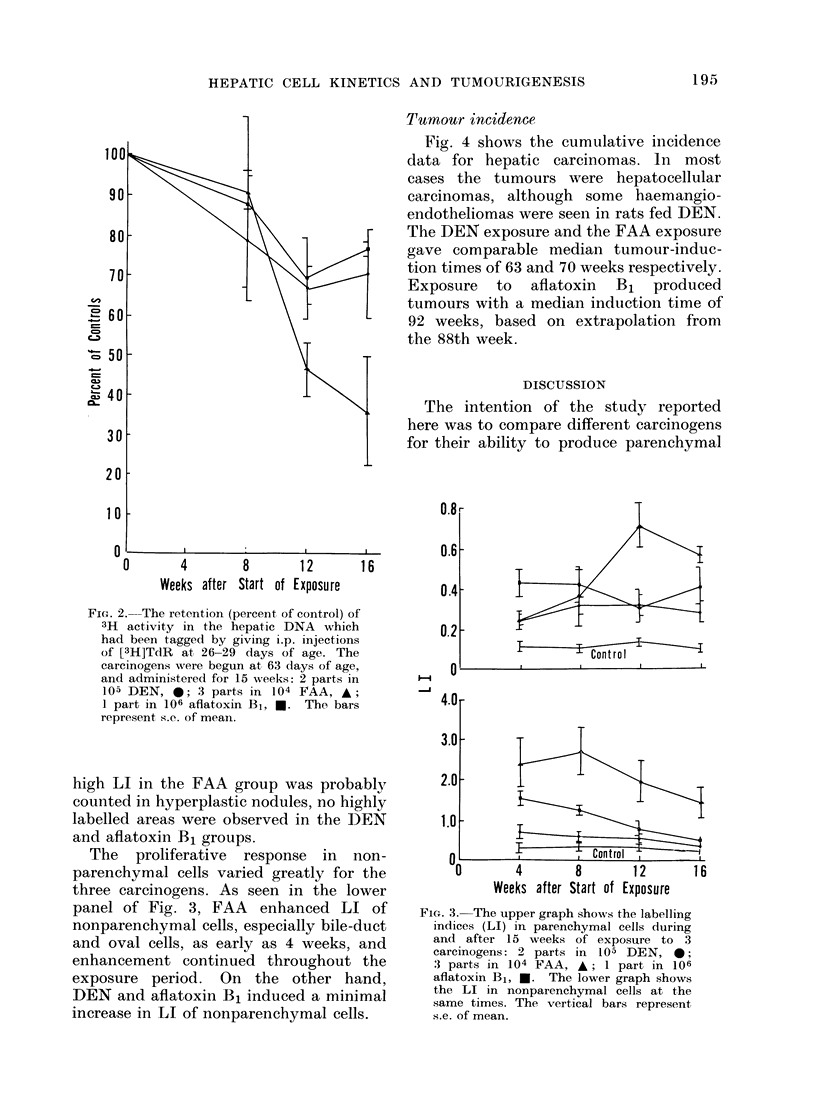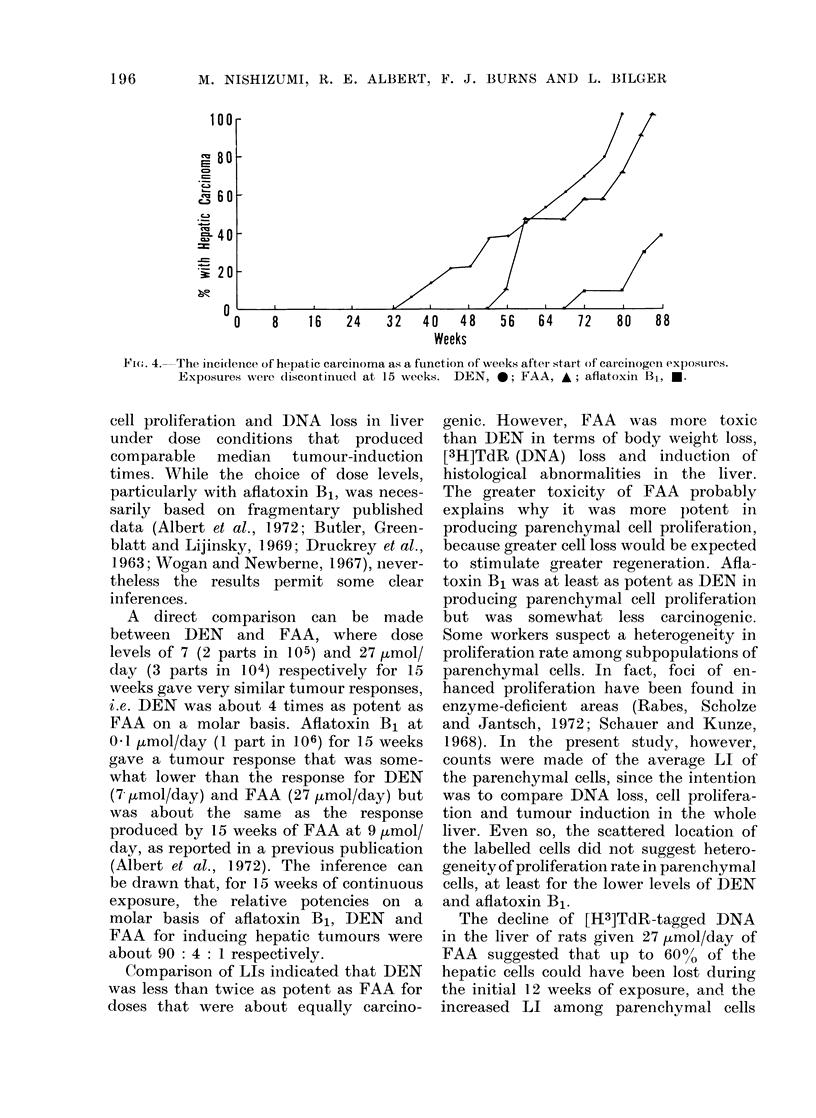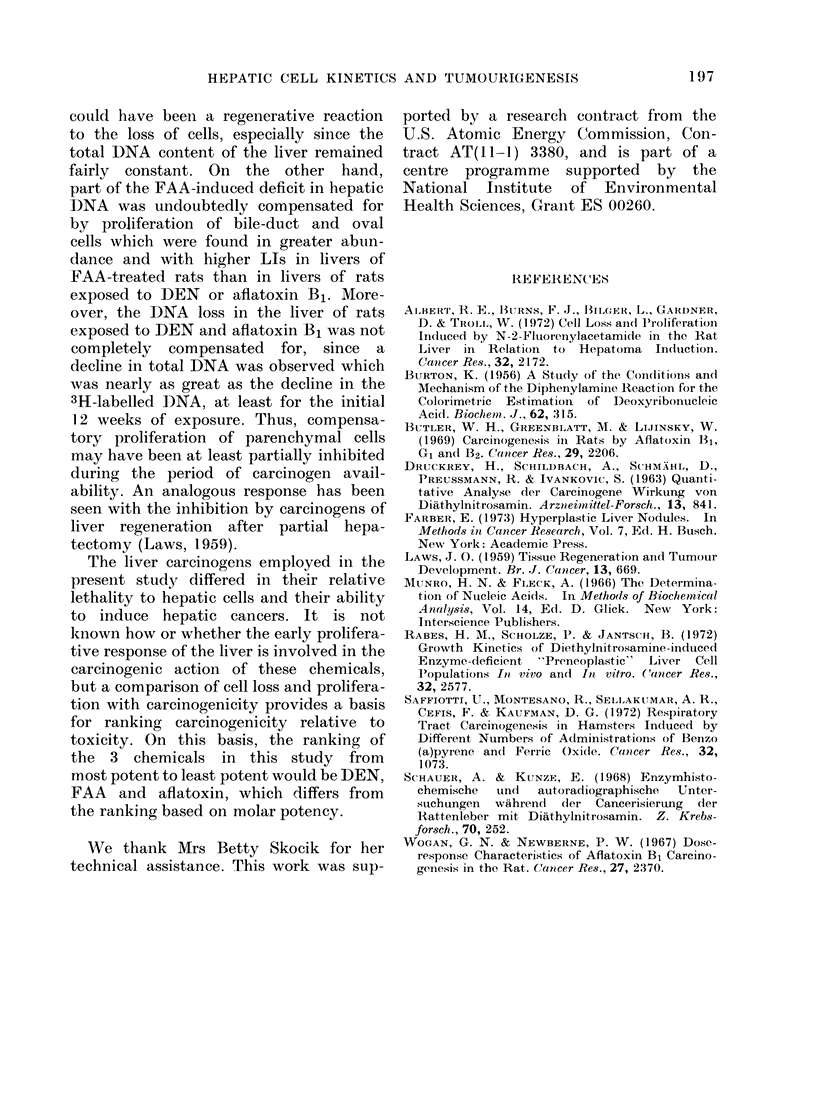# Hepatic cell loss and proliferation induced by N-2-fluorenylacetamide, diethylnitrosamine, and aflatoxin B1 in relation to hepatoma induction.

**DOI:** 10.1038/bjc.1977.177

**Published:** 1977-08

**Authors:** M. Nishizumi, R. E. Albert, F. J. Burns, L. Bilger

## Abstract

Three hepatic carcinogens (aflatoxin B1, diethylnitrosamine (DEN) and N-2-fluorenylacetamide (FAA)) were compared for carcinogenicity, early cell toxicity and parenchymal cell proliferation. The carcinogens were administered to rats for 15 weeks as follows: aflatoxin B1, 1 in 10(6) in pelleted food; DEN, 2 in 10(5) in drinking water; FAA, 3 in 10(4) in pelleted food. The loss of prelabelled DNA and the [H3] TdR pulse-labelling indices (LI) of parenchymal and nonparenchymal cells were determined at various times during the period of carcinogen availability. On a molar basis, aflatoxin B1 was 90 times as carcinogenic as FAA and 24 times as carcinogenic as DEN. However, for about equal magnitudes of hepatic cell proliferation and loss, aflatoxin B1 was the least potent carcinogen. For a given level of carcinogenicity, FAA was more potent than DEN in causing loss of hepatic DNA and in increasing the parenchymal cell labelling index. DEN and aflatoxin B1 produced about the same degree of DNA loss and parenchymal cell labelling, but the former was a more potent carcinogen. When carcinogenicity was compared for approximately equal levels of early hepatic cell destruction and proliferation, the 3 chemicals in the present study could be ranked in descending order of potency as DEN, FAA and aflatoxin B1.


*Br. J. Cancer* (1977) **36**, 192.

### HEPATIC CELL LOSS AND PROLIFERATION INDUCED BY N-2-FLUORENYLACETAMIDE, DIETHYLNITROSAMINE, AND AFLATOXIN $B_1$ IN RELATION TO HEPATOMA INDUCTION

M. NISHIZUMI*, R. E. ALBERT, F. J. BURNS, and L. BILGER

*From the Institute of Environmental Medicine, New York University Medical Center,* 550 *First Avenue, New York, N.Y.* 10016, *U.S.A.*

Received 26 August 1976  Accepted 6 April 1977

**Summary.—Three hepatic carcinogens (aflatoxin $B_1$, diethylnitrosamine (DEN) and N-2-fluorenylacetamide (FAA)) were compared for carcinogenicity, early cell toxicity and parenchymal cell proliferation. The carcinogens were administered to rats for 15 weeks as follows: aflatoxin $B_1$, 1 in $10^6$ in pelleted food; DEN, 2 in $10^5$ in drinking water; FAA, 3 in $10^4$ in pelleted food. The loss of prelabelled DNA and the [$H^3$] TdR pulse-labelling indices (LI) of parenchymal and nonparenchymal cells were determined at various times during the period of carcinogen availability. On a molar basis, aflatoxin $B_1$ was 90 times as carcinogenic as FAA and 24 times as carcinogenic as DEN. However, for about equal magnitudes of hepatic cell proliferation and loss, aflatoxin $B_1$ was the least potent carcinogen. For a given level of carcinogenicity, FAA was more potent than DEN in causing loss of hepatic DNA and in increasing the parenchymal cell labelling index. DEN and aflatoxin $B_1$ produced about the same degree of DNA loss and parenchymal cell labelling, but the former was a more potent carcinogen. When carcinogenicity was compared for approximately equal levels of early hepatic cell destruction and proliferation, the 3 chemicals in the present study could be ranked in descending order of potency as DEN, FAA and aflatoxin $B_1$.**

SEVERAL studies have indicated that hepatocarcinogens such as dimethylaminoazobenzene and its derivative, N-2-fluorenylacetamide (FAA), thioacetamide and diethylnitrosamine (DEN) enhance the proliferative activity of hepatic cells in the early stage of carcinogenesis. A high cell proliferation rate has also been reported in the hyperplastic liver nodules which are considered to be possible precursors of hepatic carcinomas (Farber, 1973).

Most carcinogens are toxic and are capable of killing cells, which in liver may stimulate proliferative regeneration. Previous studies in this laboratory (Albert *et al.*, 1972) showed that 0·03% dietary FAA induced a marked hepatic DNA loss, followed by the formation of regenerative and hyperplastic nodules which exhibited a high rate of cell proliferation.

The experiment reported here compared hepatic DNA loss and cell proliferation in precancerous liver to subsequent hepatic carcinoma incidence, for different types of hepatocarcinogens. Of particular interest was the degree of similarity amongst the 3 carcinogens in the comparative potency for inducing tumours and proliferative effects.

### MATERIALS AND METHODS

*Animals and carcinogens.*—A total of 252 male albino rats of the Charles River (CD) strain, obtained as weanlings, were used for this experiment. They were divided into 2 control and 6 experimental groups, according

* Present address: Department of Public Health, Faculty of Medicine, Kyushu University, Fukuoka 812, Japan.

to the kind of carcinogen administered, and the time of tagging with tritiated thymidine ([$^3$H]TdR: 12 Ci/mmol; Schwarz Biochemical, Orangeburg, N.Y.). Three parts in $10^4$ FAA (Eastman Organic Chemicals, Rochester, N.Y.) and 1 part in $10^6$ aflatoxin $B_1$ (Calbiochem, San Diego, California) were administered in a mixed pellet-feed of Bio Serve Chow (Frenchtown, N.J.). Two parts/$10^5$ DEN (Eastman Organic Chemicals, Rochester, N.Y.) was given in drinking water dispensed from light-tight containers. Carcinogen exposures were begun at 63 days of age (325 g) and continued for 15 weeks. The animals were housed 2 per cage and body weights were recorded once a week throughout the experiment. Consumption of feed during the exposure period was 25 g/rat/day for control and DEN-treated rats and 25% less for FAA-treated rats. No differences were noted in the consumption of drinking water in any of the treated or control groups.

*Cell kinetics study.*—Autoradiography and DNA assay of the liver for 5–6 rats in each group were performed at 4, 8, 12 and 16 weeks after the start of carcinogen exposure. The extent of DNA loss was estimated by the loss of $^3$H activity from the total hepatic DNA in rats tagged with [$^3$H]TdR as weanlings. In order to tag the DNA, 20 $\mu$Ci of [$^3$H]TdR per rat was injected i.p. on each of 4 successive days, beginning at 26 days of age. The hepatic DNA was extracted by means of a modified Schmidt–Thannhauser procedure (Munro and Fleck, 1966) and the amount of DNA was estimated on the basis of Burton's reaction (Burton, 1956). The $^3$H in the DNA was measured by liquid scintillation counting (Nuclear Chicago, Des Plains, Ill.).

The degree of hepatic cell proliferation was assessed in rats previously unexposed to [$^3$H]TdR by giving single i.p. injections of 1·0 $\mu$Ci/g [$^3$H]TdR 1·5 h before killing and determining the labelling index (LI) of hepatocytes autoradiographically. Autoradiographs were prepared as described before (Albert *et al.*, 1972). LI determinations and counts of labelled parenchymal and nonparenchymal cells were made on each autoradiograph, on 80 consecutive fields of 0·04 mm$^2$, roughly in the centre of sections. The number of parenchymal and nonparenchymal cells was determined on 20 consecutive 0·01-mm$^2$ fields also in the centre of the same sections. Nuclei with grain counts $>5$ were counted as labelled, and the LI was based on the count of 4000–6000 cells for parenchymal cells and about 2000 cells for nonparenchymal cells.

*Tumour incidence study.*—After carcinogen exposure, 16 rats in each treatment group were kept on control diet to determine the tumour incidence. The rats were examined by autopsy if found dead or killed because of their moribund condition, and the livers were examined histologically. Tumour formation was expressed in terms of the cumulative mortality-corrected incidence by a life-table method (Saffiotti *et al.*, 1972).

### RESULTS

### *Body weight, liver weight and hepatic DNA*

Fig. 1 shows the body weight, the ratio of liver weight to body weight, and the total hepatic DNA, as functions of time in weeks after the start of the exposures. Inhibition of body growth during the carcinogen exposure was observed in the rats fed FAA, but not in other groups. The liver-to-body ratio was also affected in the FAA group. No decrease in total hepatic DNA was noted in the FAA group through the exposure period; indeed there was a slight increase at 12 and 16 weeks. Aflatoxin $B_1$ produced a small decrease in total hepatic DNA beginning at 8 weeks, which persisted through 16 weeks.

### *Gross and histological findings of the liver*

The liver of rats fed FAA showed marked cirrhotic changes with nodule formation by 12 weeks, but exposure to DEN or aflatoxin $B_1$ induced none of these changes by the end of the 16-week period of exposure. Later, many nodules were found in the non-tumourous portions of the liver in rats exposed to FAA that died with hepatic tumours, but no macroscopic nodules were found in rats dying with hepatic tumours who had received aflatoxin $B_1$ or DEN.

Histological findings in three experimental groups at 8 and 16 weeks are shown in the Table. Vacuolation of

FIG. 1.—The body weight, the ratio of liver weight to body weight, and the total hepatic DNA, as percentage of corresponding control, at various times during and after 15 weeks of exposure to the following carcinogens: 2 parts in $10^5$ DEN ●; 3 parts in $10^4$ FAA, ▲; 1 part in $10^6$ aflatoxin $B_1$, ■. Each point represents the mean for 10 to 12 rats.

cytoplasm in hepatocytes, and proliferation of bile-duct and oval cells began to appear as early as 4 weeks after the start of exposure to FAA. In this group, vacuolar changes of hepatocytes were found throughout the lobules at 8 weeks, and there after hyperplastic nodules appeared and increased in number progressively at 12 and 16 weeks. After several months, biliary cysts as well as hepatocellular carcinoma were frequently observed in the rats exposed to FAA. On the other hand, rats receiving DEN and aflatoxin $B_1$ showed, at 12 and 16 weeks, only small foci consisting of hepatocytes with vacuolated cytoplasm and slight nuclear irregularities, including a few large nuclei. Neither hyperplastic nodules nor proliferation of bile-duct and oval cells were seen during the exposure in rats that received DEN or aflatoxin $B_1$.

*Loss of hepatic DNA*

Fig. 2 shows the relative amount of $^3H$ activity in the hepatic DNA of rats tagged with [$^3H$]TdR as weanlings. The activity of controls was essentially constant for the three sampling times of 8, 12, and 16 weeks. A decrease of $^3H$ in the DNA of the FAA group reached a level of about 40% of controls by 16 weeks, while the other two groups (DEN and aflatoxin $B_1$) showed a smaller decrease, to a level of about 70% of controls at 12 to 16 weeks. The relatively rapid decrease of $^3H$ activity in the DNA of the FAA group after 8 weeks occurred in association with the appearance of hyperplastic nodules.

*Hepatic cell proliferation*

As seen in the upper panel of Fig. 3, a maximum LI of the parenchymal cells (0·7%) was obtained in the FAA group at 12 weeks, while the maximum LIs for the aflatoxin $B_1$ (0·4%) and the DEN (0·3%) occurred earlier. Although the

TABLE.—*Histopathological Changes of the Liver at* 8 *and* 16 *Weeks*

| Carcinogen | Bile-duct and oval cell proliferation | | Cholangio-fibrosis | | Foci of vacuolated hepatocytes | | Nuclear irregularities | | Hyperplastic nodules | |
|---|---|---|---|---|---|---|---|---|---|---|
| | 8 wk | 16 wk | 8 wk | 16 wk | 8 wk | 16 wk | 8 wk | 16 wk | 8 wk | 16 wk |
| 3 parts in $10^4$ FAA | ++ | +++ | ± | ++ | ++ | ++ | — | + | ± | +++ |
| 2 parts in $10^5$ DEN | — | — | — | — | — | + | — | ± | — | — |
| 1 part in $10^6$ Aflatoxin $B_1$ | — | — | — | — | — | + | — | ± | — | — |

—, absent; ±, rare; +, quite frequent; ++, frequent; +++, very frequent.

FIG. 2.—The retention (percent of control) of $^{3}H$ activity in the hepatic DNA which had been tagged by giving i.p. injections of [$^{3}H$]TdR at 26–29 days of age. The carcinogens were begun at 63 days of age, and administered for 15 weeks: 2 parts in $10^5$ DEN, ●; 3 parts in $10^4$ FAA, ▲; 1 part in $10^6$ aflatoxin $B_1$, ■. The bars represent s.e. of mean.

high LI in the FAA group was probably counted in hyperplastic nodules, no highly labelled areas were observed in the DEN and aflatoxin $B_1$ groups.

The proliferative response in nonparenchymal cells varied greatly for the three carcinogens. As seen in the lower panel of Fig. 3, FAA enhanced LI of nonparenchymal cells, especially bile-duct and oval cells, as early as 4 weeks, and enhancement continued throughout the exposure period. On the other hand, DEN and aflatoxin $B_1$ induced a minimal increase in LI of nonparenchymal cells.

*Tumour incidence*

Fig. 4 shows the cumulative incidence data for hepatic carcinomas. In most cases the tumours were hepatocellular carcinomas, although some haemangioendotheliomas were seen in rats fed DEN. The DEN exposure and the FAA exposure gave comparable median tumour-induction times of 63 and 70 weeks respectively. Exposure to aflatoxin $B_1$ produced tumours with a median induction time of 92 weeks, based on extrapolation from the 88th week.

### DISCUSSION

The intention of the study reported here was to compare different carcinogens for their ability to produce parenchymal

FIG. 3.—The upper graph shows the labelling indices (LI) in parenchymal cells during and after 15 weeks of exposure to 3 carcinogens: 2 parts in $10^5$ DEN, ●; 3 parts in $10^4$ FAA, ▲; 1 part in $10^6$ aflatoxin $B_1$, ■. The lower graph shows the LI in nonparenchymal cells at the same times. The vertical bars represent s.e. of mean.

FIG. 4.—The incidence of hepatic carcinoma as a function of weeks after start of carcinogen exposures. Exposures were discontinued at 15 weeks. DEN, ●; FAA, ▲; aflatoxin $B_1$, ■.

cell proliferation and DNA loss in liver under dose conditions that produced comparable median tumour-induction times. While the choice of dose levels, particularly with aflatoxin $B_1$, was necessarily based on fragmentary published data (Albert *et al.*, 1972; Butler, Greenblatt and Lijinsky, 1969; Druckrey *et al.*, 1963; Wogan and Newberne, 1967), nevertheless the results permit some clear inferences.

A direct comparison can be made between DEN and FAA, where dose levels of 7 (2 parts in $10^5$) and 27 μmol/day (3 parts in $10^4$) respectively for 15 weeks gave very similar tumour responses, *i.e.* DEN was about 4 times as potent as FAA on a molar basis. Aflatoxin $B_1$ at 0·1 μmol/day (1 part in $10^6$) for 15 weeks gave a tumour response that was somewhat lower than the response for DEN (7 μmol/day) and FAA (27 μmol/day) but was about the same as the response produced by 15 weeks of FAA at 9 μmol/day, as reported in a previous publication (Albert *et al.*, 1972). The inference can be drawn that, for 15 weeks of continuous exposure, the relative potencies on a molar basis of aflatoxin $B_1$, DEN and FAA for inducing hepatic tumours were about 90 : 4 : 1 respectively.

Comparison of LIs indicated that DEN was less than twice as potent as FAA for doses that were about equally carcinogenic. However, FAA was more toxic than DEN in terms of body weight loss, [$^3$H]TdR (DNA) loss and induction of histological abnormalities in the liver. The greater toxicity of FAA probably explains why it was more potent in producing parenchymal cell proliferation, because greater cell loss would be expected to stimulate greater regeneration. Aflatoxin $B_1$ was at least as potent as DEN in producing parenchymal cell proliferation but was somewhat less carcinogenic. Some workers suspect a heterogeneity in proliferation rate among subpopulations of parenchymal cells. In fact, foci of enhanced proliferation have been found in enzyme-deficient areas (Rabes, Scholze and Jantsch, 1972; Schauer and Kunze, 1968). In the present study, however, counts were made of the average LI of the parenchymal cells, since the intention was to compare DNA loss, cell proliferation and tumour induction in the whole liver. Even so, the scattered location of the labelled cells did not suggest heterogeneity of proliferation rate in parenchymal cells, at least for the lower levels of DEN and aflatoxin $B_1$.

The decline of [$H^3$]TdR-tagged DNA in the liver of rats given 27 μmol/day of FAA suggested that up to 60% of the hepatic cells could have been lost during the initial 12 weeks of exposure, and the increased LI among parenchymal cells could have been a regenerative reaction to the loss of cells, especially since the total DNA content of the liver remained fairly constant. On the other hand, part of the FAA-induced deficit in hepatic DNA was undoubtedly compensated for by proliferation of bile-duct and oval cells which were found in greater abundance and with higher LIs in livers of FAA-treated rats than in livers of rats exposed to DEN or aflatoxin $B_1$. Moreover, the DNA loss in the liver of rats exposed to DEN and aflatoxin $B_1$ was not completely compensated for, since a decline in total DNA was observed which was nearly as great as the decline in the $^3$H-labelled DNA, at least for the initial 12 weeks of exposure. Thus, compensatory proliferation of parenchymal cells may have been at least partially inhibited during the period of carcinogen availability. An analogous response has been seen with the inhibition by carcinogens of liver regeneration after partial hepatectomy (Laws, 1959).

The liver carcinogens employed in the present study differed in their relative lethality to hepatic cells and their ability to induce hepatic cancers. It is not known how or whether the early proliferative response of the liver is involved in the carcinogenic action of these chemicals, but a comparison of cell loss and proliferation with carcinogenicity provides a basis for ranking carcinogenicity relative to toxicity. On this basis, the ranking of the 3 chemicals in this study from most potent to least potent would be DEN, FAA and aflatoxin, which differs from the ranking based on molar potency.

We thank Mrs Betty Skocik for her technical assistance. This work was supported by a research contract from the U.S. Atomic Energy Commission, Contract AT(11–1) 3380, and is part of a centre programme supported by the National Institute of Environmental Health Sciences, Grant ES 00260.

### REFERENCES

Albert, R. E., Burns, F. J., Bilger, L., Gardner, D. & Troll, W. (1972) Cell Loss and Proliferation Induced by N-2-Fluorenylacetamide in the Rat Liver in Relation to Hepatoma Induction. *Cancer Res.*, **32**, 2172.

Burton, K. (1956) A Study of the Conditions and Mechanism of the Diphenylamine Reaction for the Colorimetric Estimation of Deoxyribonucleic Acid. *Biochem. J.*, **62**, 315.

Butler, W. H., Greenblatt, M. & Lijinsky, W. (1969) Carcinogenesis in Rats by Aflatoxin $B_1$, $G_1$ and $B_2$. *Cancer Res.*, **29**, 2206.

Druckrey, H., Schildbach, A., Schmähl, D., Preussmann, R. & Ivankovic, S. (1963) Quantitative Analyse der Carcinogene Wirkung von Diäthylnitrosamin. *Arzneimittel-Forsch.*, **13**, 841.

Farber, E. (1973) Hyperplastic Liver Nodules. In *Methods in Cancer Research*, Vol. 7, Ed. H. Busch. New York: Academic Press.

Laws, J. O. (1959) Tissue Regeneration and Tumour Development. *Br. J. Cancer*, **13**, 669.

Munro, H. N. & Fleck, A. (1966) The Determination of Nucleic Acids. In *Methods of Biochemical Analysis*, Vol. 14, Ed. D. Glick. New York: Interscience Publishers.

Rabes, H. M., Scholze, P. & Jantsch, B. (1972) Growth Kinetics of Diethylnitrosamine-induced Enzyme-deficient "Preneoplastic" Liver Cell Populations *In vivo* and *In vitro*. *Cancer Res.*, **32**, 2577.

Saffiotti, U., Montesano, R., Sellakumar, A. R., Cefis, F. & Kaufman, D. G. (1972) Respiratory Tract Carcinogenesis in Hamsters Induced by Different Numbers of Administrations of Benzo (a)pyrene and Ferric Oxide. *Cancer Res.*, **32**, 1073.

Schauer, A. & Kunze, E. (1968) Enzymhistochemische und autoradiographische Untersuchungen während der Cancerisierung der Rattenleber mit Diäthylnitrosamin. *Z. Krebsforsch.*, **70**, 252.

Wogan, G. N. & Newberne, P. W. (1967) Dose-response Characteristics of Aflatoxin $B_1$ Carcinogenesis in the Rat. *Cancer Res.*, **27**, 2370.